# Bacterial-Specific Induction of Inflammatory Cytokines Significantly Decreases upon Dual Species Infections of Implant Materials with Periodontal Pathogens in a Mouse Model

**DOI:** 10.3390/biomedicines10020286

**Published:** 2022-01-26

**Authors:** Muhammad Imran Rahim, Andreas Winkel, Alexandra Ingendoh-Tsakmakidis, Stefan Lienenklaus, Christine S. Falk, Michael Eisenburger, Meike Stiesch

**Affiliations:** 1 Lower Saxony Centre for Biomedical Engineering, Implant Research and Development (NIFE), Department of Prosthetic Dentistry and Biomedical Materials Science, Hannover Medical School, 30625 Hannover, Germany; Winkel.Andreas@mh-hannover.de (A.W.); alexandra.ingendoh@protonmail.com (A.I.-T.); Eisenburger.Michael@mh-hannover.de (M.E.); Stiesch.Meike@mh-hannover.de (M.S.); 2 Institute of Laboratory Animal Science, Hannover Medical School, 30625 Hannover, Germany; Lienenklaus.Stefan@mh-hannover.de; 3 Institute of Transplant Immunology, Hannover Medical School, 30625 Hannover, Germany; Falk.Christine@mh-hannover.de

**Keywords:** cytokine, fibroblasts, mouse model, biomaterial-associated infections, periodontal pathogens

## Abstract

Cytokine profiles are often perturbed after infections of medical implants. With a non-invasive in vivo imaging system, we report in a mouse model that interferon expression after infection of subcutaneous implants with *Streptococcus oralis*, *Aggregatibacter actinomycetemcomitans*, *Porphyromonas gingivalis*, and *Treponema denticola* (alone or as a combination) was species-specific, persisted longer in the presence of implants, and notably decreased upon dual species infections. This type I interferon expression disappeared within two weeks; however, histology of implant–tissue interface indicated high recruitment of immune cells even after three weeks. This was suggestive that biomaterial-associated infections could have prolonged effects, including the systemic stimulation of inflammatory cytokines. The present study investigated the systemic impact of this chronic peri-implant inflammation on the systemic expression of inflammatory cytokines (23) using a multiplex assay. Initially, the cytokine measurement in murine fibroblasts exposed to periodontal pathogens remained limited to the expression of five cytokines, namely, IL-6, G-CSF, CXCL-1/KC, MCP-1 (MCAF), and IL-12 (p40). The systemic determination of cytokines in mice increased to 19 cytokines (IL-1α, IL-2, IL-3, IL-5, IL-6, IL-9, IL-12 (p40), IL-12 (p70), IL-13, IL-17A, CCL-11/Eotaxin, G-CSF, IFN-γ, CXCL1/KC, MCP-1 (MCAF), MIP-1α/CCL3, MIP-1β/CCL4, CCL5/RANTES, and TNF-α). Systemic induction of cytokines was species-specific in the mouse model. The cytokine induction from infected implants differed significantly from sole tissue infections and sterile implants. Notably, systemic cytokine induction decreased after infections with dual species compared to single species infections. These findings describe the systemic effect of chronic peri-implant inflammation on the systemic induction of inflammatory cytokines, and this effect was strongly correlated to the type and composition of initial infection. Systemic modulations in cytokine expression upon dual species infections exhibit an exciting pattern that might explain the complications associated with biomaterial-related infection in patients. Moreover, these findings validate the requirement of multispecies infections for pre-clinical studies involving animal models.

## 1. Introduction

Dental implants are used to restore masticatory function in partially or fully edentulous patients with a high success rate [1]. In diseased situations, implants can be colonized by infectious bacterial species and subsequent formation of recalcitrant biofilms can occur [2]. The pathogenesis of peri-implant diseases largely attributes to high inflammation resulting from interactions between tissue, implant material, and bacterial factors [3]. In the course of these inflammatory events, cytokines play critical role, and an imbalanced cytokine secretion may incur tissue destruction at the inflamed implant site and disease progression [4,5]. Stimulation in the secretion of inflammatory cytokines in the host was strongly correlated to the type of pathogen [6]. Recent studies have indicated a strong association of chronic inflammation to the type of bacteria related to peri-implant diseases [7,8,9]. Some inflammatory cytokines, including IL-1β, IL-6, IL-8, and TNF-α, were frequently measured during biomaterial-related infections [10]. The secretion of pro-inflammatory cytokines was shown to be critical in the pathogenesis of implant-related infections. For example, human and experimental animal models studies reported a critical role of inflammatory cytokine expressions and their receptors in alveolar bone loss [11]. The biofilm formation around dental implants includes diverse bacterial species comprising commensal streptococci as initial colonizers [12,13,14]. This initial accumulation by streptococci triggers the host for paracrine and autocrine secretion of chemical cytokines and growth factors at implantation sites in a homeostatic balance with the host immune system [11]. Towards diseased phase, a secondary accumulation of virulent species such as *P. gingivalis* or *T. denticola* increases and triggers high inflammation with uncontrolled secretion of inflammatory cytokines [15].

Biomaterial-associated infections show high infiltration and prolonged persistence of host immune cells, suggesting that these infections may be a more important inducer of inflammatory reactions [16,17]. Elevated secretions of inflammatory cytokines such as IL-1, IL-6, and IL-10 remain essential markers of implant-associated inflammatory events [18]. These dysregulations in the inflammatory cytokine expressions could be influenced by various factors such as the type of pathogens, inter-bacterial interactions, and the presence of implant material. In the latest study, a non-invasive in vivo imaging method was established in transgenic mice to monitor the status of type I interferon-β after infection of the tissue and subcutaneous implants with different periodontal pathogens [19]. The interferon expression kinetics was bacterial species-specific, decreased for dual species infections, and remained prolonged around infected tissue in the presence of implants compared to tissue infections without implants. The imaging of type I interferon expression was possible for two weeks, and then it disappeared completely. The histology of infected peri-implant tissue indicated high recruitment of immune cells at the tissue–implant interface. This recruitment of inflammatory cells was mild around sterile implants or infected tissues without implants. It was therefore hypothesized that this intense tissue inflammation could modulate the expression of inflammatory cytokines. Serum was collected from mice to satisfy this hypothesis, and multiplex cytokine analysis was performed to measure 23 inflammatory cytokines. Since fibroblasts are the first cells to contact immediately with implanted materials or injected pathogens [20], cells were exposed directly to four periodontal pathogens as single or as multispecies to monitor the sensitivity of cells and the induction of inflammatory cytokines.

## 2. Materials and Methods

### 2.1. Bacterial Cultivation

*Streptococcus oralis* (ATCC 9811, American Type Culture Collection, Manassas, VA, USA), *Aggregatibacter actinomycetemcomitans* (DSM 11123, German Collection of Microorganisms and Cell Cultures, Braunschweig, Germany), and *Porphyromonas gingivalis* (DSM 20709) were cultured on tryptone soy agar (TSA) or fastidious anaerobe agar (FAA) plates supplemented with 5% sheep blood at 37 °C under anaerobic conditions (80% N_2_, 10% H_2_, 10% CO_2_). Few colonies of *S. oralis* were inoculated into tryptone soy broth (TSB) (Oxoid Limited, Hamsphire, United Kingdom) supplemented with 10% yeast extract (Carl Roth GmbH + Co. KG, Karlsruhe, Germany) and 50 mM glucose (Carl Roth GmbH + Co. KG, Karlsruhe, Germany) at 37 °C under constant shaking. A few colonies of *A. actinomycetemcomitans* or *P. gingivalis* were inoculated overnight into brain heart infusion medium (BHI; Oxoid, Wesl, Germany) supplemented with 10 μg/mL vitamin K (Roth, Karlsruhe, Germany) under anaerobic conditions [21]. *Treponema denticola* (DSM 14222) cultures were prepared in new oral spirochete (NOS) medium at 37 °C for 72 h under static anaerobic conditions [22].

### 2.2. Co-Cultivation of Murine Fibroblasts with Bacteria

Murine fibroblasts (NIH3T3) were cultured in DMEM supplemented with 10% FCS under standard cell culture conditions [23]. Confluent cells were washed with PBS, suspended in DMEM without antibiotics, and then seeded on glass coverslips 10^6^ cells per specimen (three biological replicates, each biological replicate was run in three technical replicates). Cells were incubated for 24 h under standard cell culture conditions and then exposed individually to 10^5^ colony-forming units of *S. oralis*, *A. actinomycetemcomitans, P. gingivalis*, or *T. denticola*. Challenge of fibroblasts with a mixture of all species was done by 10^5^ colony-forming units of each species. Infected cells were incubated for 24 h under standard cell culture conditions. After 24 h, supernatants were collected, centrifuged, and stored at −80 °C for multiplex assay. For confocal microscopy, adherent cells were treated for 30 min with SYTO^®^9 and propidium iodide (1:1000 dilutions in PBS) (LIVE/DEAD^®^Backlight^TM^ Bacterial Viability Kit, Life Technologies, Carlsbad, CA, USA) [21,24]. Stained cells were imaged with confocal laser scanning microscope (CLSM) (SP-8, Leica Microsystems, Wetzlar, Germany). SYTO^®^9 signals were measured with multi-wavelength argon laser (excitation wavelength 488 nm) and an emission range of 500–550 nm.

### 2.3. Preparation of Titanium Implants

**Figure 1:**
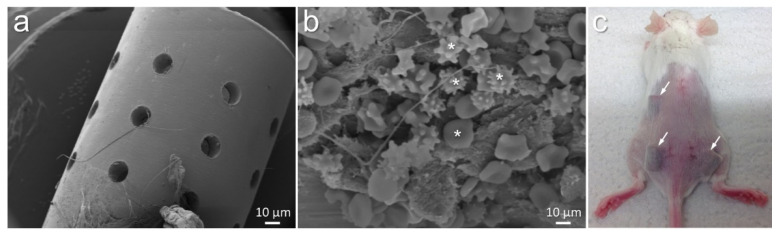
Morphology of titanium cylinders designed for implantation into mice (**a**). Implants allowed accumulation of various host cells visible after 21 days of implantation ((**b**), white asterisks). Mouse showing the impression of subcutaneously implanted titanium cylinders ((**c**), white arrows indicate implanted titanium).

Implantable cylinders with 4.5 mm diameter were fabricated from grade 4 titanium (L.Klein SA, Biel, Switzerland). These implants with 7 mm length, 3.3 mm diameter, and 24 pores (0.5 mm each) were manufactured at Central Research Devices Service Unit, Hannover Medical School, Germany (Figure 1a) [19]. Such porous cylinders were expected to provide safe niches to the injected bacteria from the invasion of host immune cells (Figure 1b). These cylinders were implanted subcutaneously into mice (Figure 1c).

### 2.4. Subcutaneous Implantations and Infections in Small Animal Model

Animal experiments were performed on female 8–12-week-old transgenic mice (C.Balb/c1-Ifnb1tm1.2Lien, bred at the Central Animal Facility, Hannover Medical School, Hannover, Germany) [19]. All animals were kept inside individually ventilated cages with optimum provision of food and water. For each infection (alone or as combination), three animals were used. All animals were first anesthetized under a sterile bench with intraperitoneal injection of 10 mg/kg ketamine (Albrecht, Germany) and 4 mg/kg xylazine (Rompun, Bayer, Leverkusen, Germany). The fur from the sites of implantations was removed with a hair trimmer (Aesculap Suhl, GmbH, Germany) and then disinfected with 70% ethanol. Three incisions (1 cm each) were created with surgical scissor and tissue forceps (Fine Science Tools, GmbH, Heidelberg, Germany). Subcutaneous pouches were created from these incisions. Cylindrical titanium implants were placed into these pouches. Wounds were closed with simple interrupted sutures (Ethicon Vicryl, Johnson & Johnson Medical GmbH). Within 30 min after the closure of surgical wounds with or without implantation, infections of 5 μL per implant with bacterial suspension (as single or dual species from 1 × 10^8^ CFU/mL) were done. Animals were then regularly observed, and blood was collected three weeks after implantation. All animal experiments were performed at Institute for Laboratory Animal Science and Central Animal facility, Hannover Medical School, according to the permission from the Lower Saxony State Office for Consumer Protection and Food Safety, Germany, with number: 33.12-42502-04-17/2580.

### 2.5. Measurement of Cytokines in Serum

After three weeks of implantation, mice were euthanized with high doses of anesthesia, and blood was collected intracardially without any coagulant. Blood samples were incubated at room temperature for 30 min and then centrifuged at 1500× *g* for 10 min at 4 °C. After centrifugation, the serum was collected and stored at −80 °C until further processing. The levels of IL-1α, IL-1β, IL-2, IL-3, IL-4, IL-5, IL-6, IL-9, IL-10, IL-12 (p40), IL-12 (p70), IL-13, IL-17A, CCL-11/Eotaxin, G-CSF, GM-CSF, IFN-γ, CXCL1/KC, MCP-1 (MCAF), MIP-1α/CCL3, MIP-1β/CCL4, CCL5/RANTES, and TNF-α in serum were determined by using Bio-Plex Pro Mouse Cytokine 23-Plex Assay (Bio-Rad, Munich, Germany).

### 2.6. Statistical Analysis

SPSS Statistics (IBM, v. 26) was used for statistical analysis. The Kruskal–Wallis *H* test, a rank-based nonparametric test, with Dunn’s post hoc method was applied to test the null hypothesis that the following group were significantly different from each other: (i) sterile implant vs. various infected implants, (ii) tissue infections vs. peri-implant tissue infections, (iii) single vs. dual species infections for both peri-implant and tissues alone. Significance values were adjusted by the Bonferroni correction for multiple tests. Symbols ***, **, *, and # indicated *p* < 0.001, *p* < 0.01, *p* < 0.05, and significant *p*-value prior to Bonferroni correction, respectively.

## 3. Results

### 3.1. Cytokines Expression in Murine Fibroblasts

**Figure 2:**
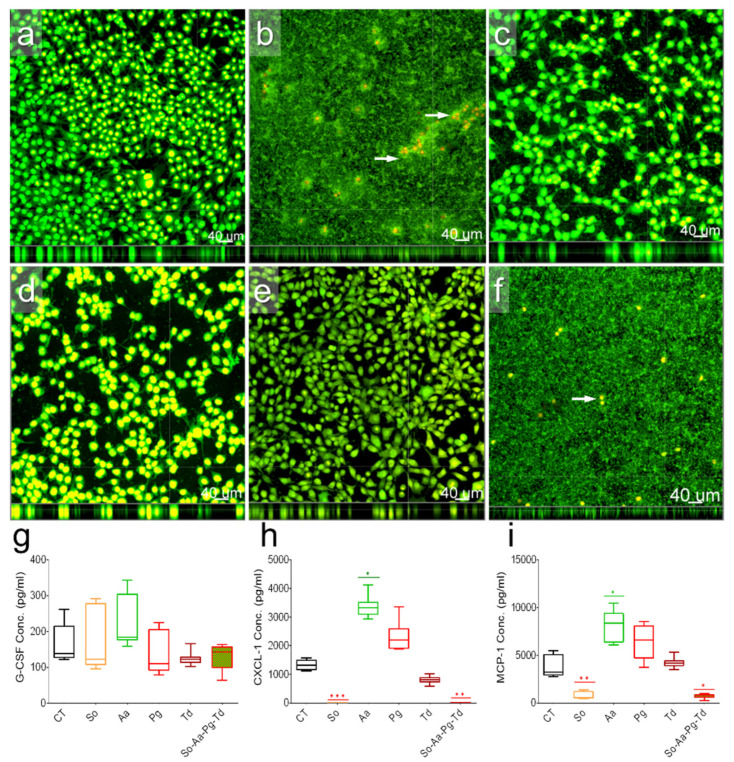
Morphology and the expression of G-CSF, CXCL-1, and MCP1 in fibroblasts. Microscopic documentation of unstimulated fibroblasts (**a**) as well as those stimulated with *S. oralis* surrounding murine fibroblasts (**b**), *A. actinomycetemcomitans* (**c**), *P. gingivalis* (**d**), and *T. denticola* (**e**), or a mixture of all four bacterial species (**f**). White arrows target remaining fibroblasts (**b**,**f**). The box and whisker graphs indicate the expression of G-CSF (**g**), CXCL-1 (**h**), and MCP-1 (**i**) in uninfected fibroblasts (CT) or after co-cultivation with *S. oralis* (So), *A. actinomycetemcomitans* (Aa), *P. gingivalis* (Pg), and *T. denticola* (Td), or a mixture of all species (So-Aa-Pg-Td). Significant changes according to the uninfected control were adjusted by Bonferroni correction for multiple tests. Symbols ***, **, and * indicate *p* < 0.001, *p* < 0.01, and *p* < 0.05, respectively.

Fibroblasts are key structural cells to contact directly with implants and invading pathogens. Multiplex cytokine analysis was performed on cell culture supernatants after exposure of murine fibroblasts to commensal *S. oralis* or pathogenic *A. actinomycetemcomitans, P. gingivalis*, or *T. denticola* or a mixture of these species. Fibroblasts without infections showed homogenous cell layer seemingly adhering well with underlying surfaces (Figure 2a). Fibroblasts exposed to *S. oralis* could not survive with faster bacterial growth (Figure 2b, white arrows). Fibroblasts co-cultured with *A. actinomycetemcomitans, P. gingivalis*, and *T. denticola* still proliferated (Figure 2c–e). Fibroblasts exposed to the mixture of four species could also not survive, whereas remaining bacterial biofilms were dominated mainly by *S. oralis* (Figure 2f). Cytokine 23-plex assay could determine the expression of only five cytokines (Figure 2g–i and Figure S2). Particularly, for fibroblasts exposed to *S. oralis* or mixed bacterial cultures, cytokine expressions were subsequently very low (Figure 2g–i and Figure S2). There seemed bacterial-specific stimulation of IL-6, IL-12 (p40), G-CSF, CXCL-1, and MCP-1 in fibroblasts exposed to *A. actinomycetemcomitans*, *P. gingivalis*, or *T. denticola* (Figure 2g–i and Figure S2). The expression of G-CSF and CXCL-1 decreased statistically non-significantly in cells exposed to *T. denticola* compared to uninfected cells (Figure 2g–i and Figure S2).

### 3.2. Systemic Analysis of Serum Interleukin

**Figure 3:**
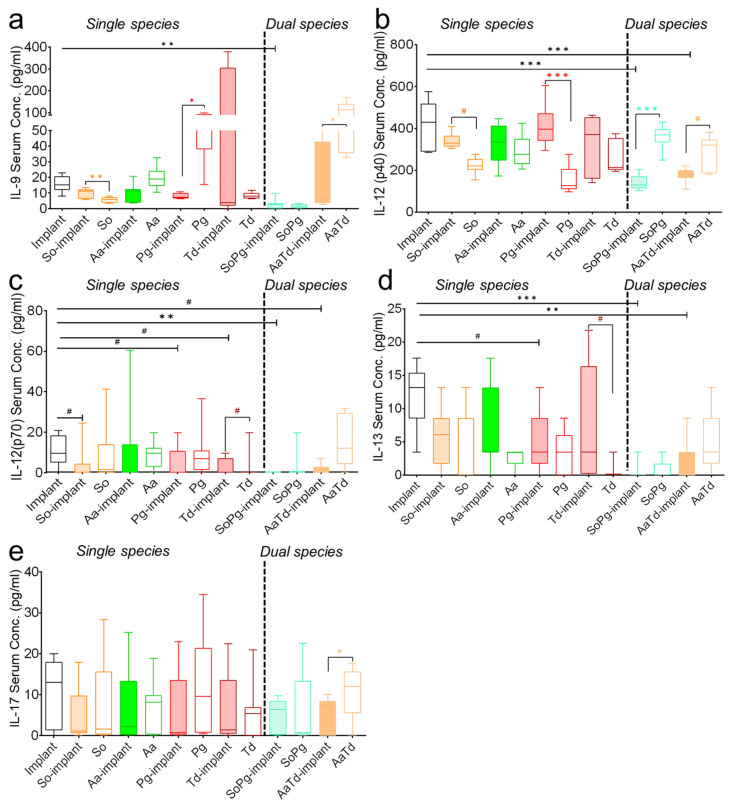
Modulations in the systemic expression of interleukins in mice. The box and whiskers graphs indicate serum interleukin expression for IL-9 (**a**), IL-12p40 (**b**), IL-12p70 (**c**), IL-13 (**d**), and IL-17 (**e**) in animals with sterile implants (empty bars), infected implants (filled colored bars), and infected tissues (empty bars with colored borders). Data were analyzed animal-wise (biological replicates (*n* = 3), technical replicates (3 each)). Significant values have been adjusted by the Bonferroni correction for multiple tests. Symbols ***, **, *, and # indicate *p* < 0.001, *p* < 0.01, *p* < 0.05, and significant *p*-value prior to Bonferroni correction, respectively, between sterile and infected implants (black symbols). Significant differences between infections in the presence and absence of implants are indicated by colored symbols in each case. Abbreviations: So (*S. oralis*), Aa (*A. actinomycetemcomitans*), Pg (*P. gingivalis*), Td (*T. denticola*).

Multiplex assay could determine an array of 19 inflammatory cytokines in mice serum including interleukin, chemokines, and growth-stimulating factors. The status of interleukin expressions from the infected implants or infected tissues without implants varied from the sterile implants (Figure 3 and Figure S3). Peri-implant infections had variable consequence in the interleukin expression than tissue infections without implants (Figure 3a and Figure S3). Expression of pro-inflammatory IL-6 was in a low detection range, but its secretion pattern was specific to the bacterial species and increased significantly (*p* = 0.0076 and 0.0058, respectively) in *S. oralis-* and *T. denticola*-infected implants compared to infected tissue without implants (Figure S3E, single species). Although in a low detection range, expression of pro-inflammatory IL-1α was specific to the bacterial species and increased in the presence of implants with *A. actinomycetemcomitans* or *P. gingivalis* compared to sterile implants (Figure S3A, single species). Dual species implant infections either with *S. oralis–P. gingivalis* or *A. actinomycetemcomitans–T. denticola* caused a significant decrease in the interleukin expression compared to single species infections or sterile implants (Figure 3 and Figure S3). Dual species infections with *S. oralis–P. gingivalis* caused significant (*p* = 0.002 and 0.004, respectively) decreases in the expression of IL-1α and IL-6 compared to sterile implants (Figure S3A,D, dual species). Serum levels of IL-12 (p40) significantly (*p* = 0.0059 and 0.0058, respectively) decreased with dual species infections compared to sterile implants (Figure 3b). IL-12 (p70) expression remained species-specific in single species infections and decreased in dual species infections (Figure 3c). The interspecies and single versus dual species differences regarding the expression of IL-17 were mild (Figure 3). Levels of IL-1β, IL-4, IL-10, and GMCSF were low or even below detection limits in either infected or sterile implants (Table S1). This analysis confirmed that implant material and dual species infections were playing an important role in the interleukin expression.

### 3.3. Systemic Analysis of Serum Chemokines

**Figure 4:**
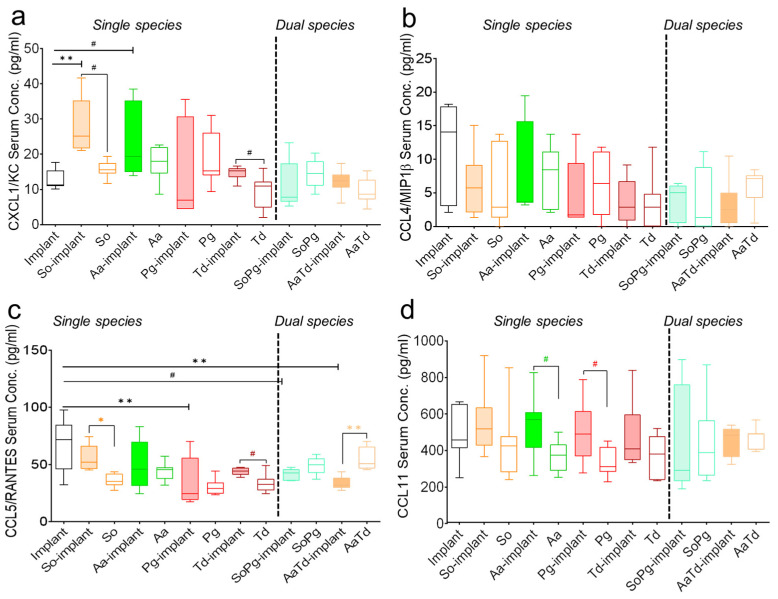
Systemic expression of chemokines in mice. Black bars represent sterile implants, filled bars indicate infected implants, and empty colored bars indicate infected tissues for CXCL1/KC (**a**), CCL4/MIP1β (**b**), CCL5/RANTES (**c**), and CCL11 (**d**). Data were analyzed animal-wise (biological replicates (*n* = 3), technical replicates (3 each)). Significant values were adjusted by the Bonferroni correction for multiple tests. Symbols **, *, and # indicate *p* < 0.01, *p* < 0.05, and significant *p*-value prior to Bonferroni correction, respectively. Black symbols represent significant differences between sterile and infected implants. Significant differences between infected implants and infected sham-operated tissues are indicated by colored symbols. Abbreviations: So (*S. oralis*), Aa (*A. actinomycetemcomitans*), Pg (*P. gingivalis*), Td (*T. denticola*).

Since chemokines are an important family of cytokines and critical mediators of host immunity, the systemic status of chemokines was monitored in the multiplex assay as well [25]. The chemokine secretions in infected tissues with or without implants changed significantly compared to sterile implants (Figure 4). Chemokine expression was bacterium specific and showed different profiles in the presence of implants. For each bacterial species, the expression of chemokines was modified by the presence of implant (Figure 4). Dual species implant infections decreased chemokine concentrations compared to corresponding single species infections (Figure 4). CXCL1/KC expression seemed dependent on *S. oralis* and *A. actinomycetemcomitans* and significantly increased (*p* = 0.0058 and 0.038, respectively) in infected implants compared to sterile implants (Figure 4a). The level of pro-inflammatory chemokine CCL3/MIP1α in all infected groups was similar to the sterile implants (Figure S2F). The expression of CCL5/RANTES decreased in all infections in the presence or absence of implants compared to sterile implants (Figure 4c). Systemic expressions of the macrophage inflammatory protein CCL4/MIP-1β was consistently low in all infected implants or tissues compared to sterile implants (Figure 4b,d). In conclusion, there seemed in majority of the cases species-specific induction of chemokines.

### 3.4. Systemic Analysis of Growth Factors and Cellular Regulators

**Figure 5:**
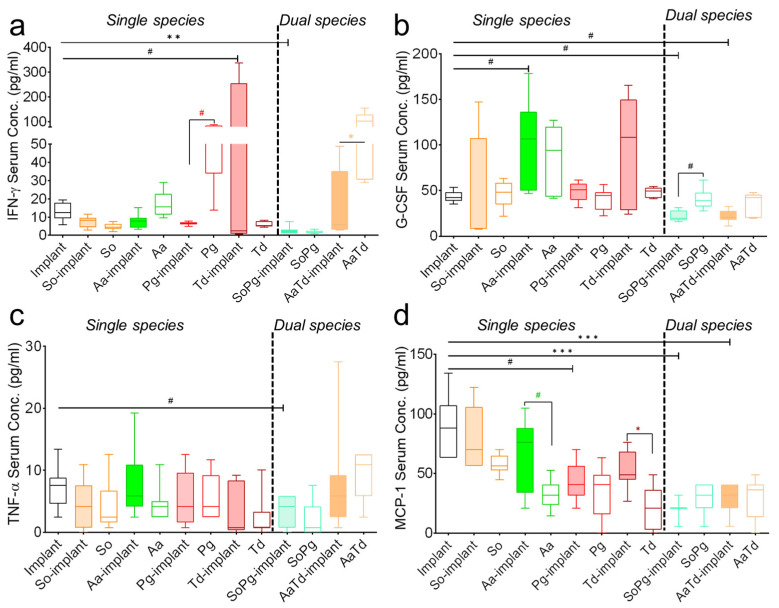
Systemic levels of inflammatory cytokines. Graphs show serum levels of IFN-γ (**a**), G-CSF (**b**), TNF-α (**c**), and CCL2/MCP-1 (**d**) 21 days after implantations and infections. Empty bars represent sterile implants, colored bars indicate infected implants, and empty bars with colored borders indicate infected tissues. Data from three independent experiments are shown. Data were analyzed animal-wise (biological replicates (*n* = 3), technical replicates (3 each)). Significant values were adjusted by the Bonferroni correction for multiple tests. Symbols ***, **, *, and # indicate *p* < 0.001, *p* < 0.01, *p* < 0.05, and significant *p*-value prior to Bonferroni correction, respectively. Black symbols represent significant differences between sterile and infected implants. Colored symbols indicate significant differences between infected implants and infected tissues in the absence of implants. Abbreviation: So (*S. oralis*), Aa (*A. actinomycetemcomitans*), Pg (*P. gingivalis*), Td (*T. denticola*).

Multiplex assay was further extended to the systemic investigation of host inflammatory responses, including interferon-gamma (IFN-γ), granulocyte colony-stimulating factor (G-CSF), tumor necrosis factor alpha (TNF-α), and monocyte chemotactic protein 1 (MCP-1) (Figure 5). Infections of implants or tissues showed the same effect in the expression of IFN-γ, G-CSF, and MCP-1 compared to sterile implants (Figure 5a). The effect of infection was clearly visible for the expression of G-CSF (Figure 5b). Serum TNF-α levels remained similar for sterile implants or after infections with implants or for infected tissues (Figure 5c). The systemic expression of MCP-1 decreased for all infections compared to sterile implants (Figure 5d). There was mild decrease in the serum levels of IFN-γ, G-CSF, CCL2/MCP-1, and IFN-γ after dual species infections compared to sterile implants (Figure 5a,b,d). To conclude, systemic expression of growth-stimulating factors was highly species-specific and decreased mostly for dual species infections.

Significant changes between two different treatments (according to the presence or absence of implants, sterile vs. infected implants with a certain bacterium or combination of bacteria) are shown in tables to present further peculiarities (Table 1 and Table 2). Infected implants caused an increase in the cytokine induction for *A. actinomycetemcomitans* compared to sterile implants while causing a decrease for *P. gingivalis* infections compared to sterile implants (Table 1). Throughout dual species implant infections with *A. actinomycetemcomitans–T. denticola* or *S. oralis*–*P. gingivalis* consistently decreased more cytokines compared to any single species implant infections (Table 1). Statistical comparison between dual species and single species infections indicated significant downregulation of cytokine after dual species infections of implants when compared to single species infections (Table S2). Statistical analysis between single species-infected implants and infected tissues without implants indicated an upregulation in the expression of cytokines. However, in *P. gingivalis*, implant infections decreased the systemic expression of four cytokines compared to tissue infections without implants (Table 2). In dual species infection, a downregulation in the cytokine expression was observed in the presence of implants.

## 4. Discussion

Infectious biofilms around dental implants cause uncontrolled cytokine secretions and are the first stage of peri-implant mucositis and peri-implantitis development [41]. In the latest finding, type I interferon-β expression around infected implants was dependent on the type of pathogen, implant material, and the mixture of two species. Notably, in-vivo expression of interferon-β decreased around implants infected with dual species compared to implant infections with the same bacteria as a single species [19,42]. While the interferon expression had entirely disappeared after three weeks, histological analysis of the infected implant–tissue interface indicated high recruitment of inflammatory cells. This recruitment of inflammatory cells at implant–tissue interfaces infected with dual species was more elevated than peri-implant tissues infected with single species. Such histological findings confirmed that a chronic inflammatory situation had developed around infected implants. The chronic inflammatory condition was not measurable by non-invasive in vivo imaging of type I interferon-β.

Fibroblasts are known to make immediate contact with implants or invading pathogens and express inflammatory cytokines [20,23]. Therefore, fibroblasts were exposed to *S. oralis* and *A. actinomycetemcomitans*, reported as initial colonizers, and *P. gingivalis* and *T. denticola*, reported as late colonizers. Four pathogens were mixed as multispecies to investigate the effect of polymicrobial biofilms, and fibroblasts were exposed. Murine fibroblasts seemed highly sensitive to direct exposure of *S. oralis* and could not proliferate. In another study that investigated the expression of IL-6, CCL-2, and CXCL-8 in human gingival epithelial cells (HGEps) and fibroblasts (HGFs) exposed indirectly to *S. oralis*, there was a variable expression of IL-6 and CCL-2 in both cell lines [43]. Notably, the expression of IL-6 significantly decreased in human gingival fibroblasts exposed to *S. oralis* while the CCL-2 showed a non-significant increase. Compared to this study, fibroblasts in the present study showed a non-significant increase in IL-6. The difference in IL-6 expression seemed mainly from a difference in the type of cells and the co-culture process. In the present study, direct exposure of murine fibroblasts to *S. oralis* damaged the cells and led to a proinflammatory situation, causing an increase in IL-6. Fibroblasts co-cultured with *A. actinomycetemcomitans*, *P. gingivalis,* and *T. denticola* managed to proliferate since these bacteria were anaerobic and could not exhibit optimum growth under standard cell culture conditions. In vitro cytokine analysis was restricted to detecting five cytokines, namely, IL-6, G-CSF, CXCL-1/KC, MCP-1 (MCAF), and IL-12 (p40). Among these detectable cytokines, *T. denticola* exposure led to a non-significant decrease in cytokine induction compared to uninfected fibroblasts. Such decrease in the cytokine production upon *T. denticola* could be related to previous observation for this bacteria to degrade cytokine for modulation in the immune responses [44,45]. Fibroblasts exposed to *P. gingivalis* showed a non-significant increase in IL-6 secretion compared to uninfected cells, and this type of trend was observed in epithelial cells, which showed high secretion of IL-1β, TNF-α, IL-6, and CXCL8/IL-8 after exposure to *P. gingivalis* [46,47]. Importantly, fibroblasts exposed to multi-species infections could not survive, and a decrease in the cytokine induction was observed. Overall, fibroblast seemed extremely sensitive to bacterial cultivations, and cytokine detection remained limited.

For in vivo evaluation, we selected a mouse model with subcutaneous implantations to investigate the detailed effect of biomaterial-associated infections by various bacterial species on the host. This sub-cutaneous mouse model allowed for easy implantation of large-sized titanium cylinders and the possibility of facilitating direct interaction of immune cells with infected implants. This subcutaneous mouse model quickly interpreted the effects of biomaterial-related infections and was less stressful for animals. The multiplex analysis in mice could measure the expression of 19 inflammatory cytokines, including interleukins, chemokines, and growth-stimulating factors. This systemic expression for most inflammatory cytokines downregulated after *P. gingivalis* or *T. denticola*, chronic inflammatory pathogens, in comparison with *A. actinomycetemcomitans*, responsible for aggressive inflammation [48,49]. The explanation could be the ability of *P. gingivalis* to secrete proteolytic enzymes that degrade cytokines and chemokines [50,51]. The majority of results indicated a decrease in the systemic cytokine induction after *T. denticola* infections with implants compared to sterile implants. The reduction in the cytokine after *T. denticola* infections could possibly be related to the ability of *T. denticola* to secrete proteinases that are known to degrade cytokines. The other reason could be associated with the limited capacity of *T. denticola* to interact with appropriate receptors for the release of cytokines [52,53]. Infections in the presence of implants showed different systemic effects in the secretions of cytokines compared to infected tissues without implants. From this observation, it can be assumed that tissues around implant materials allowed prolonged bacterial survival even in the presence of the host immune system. As shown in Table 2, *A. actinomycetemcomitans* infection of implants caused a significant increase in the systemic expression of IL-2, IL-3, IL-5, CCL-3/MIP1α, and CCL2/MCP-1 compared to tissue infections without implants. *T. denticola* single species implant infections systemically increased IL-6, IL-13, CXCL1/KC, CCL-3/MIP1α, and CCL2/MCP compared to tissue infection without the implant. *Staphylococcus epidermidis* infections of implants in tissues increased localized secretion of interleukin-1 [54,55]. Moreover, IL-1 and TNF-α production in human monocytes increased by the addition of LPS in the presence of biomaterials [56]. Pro-inflammatory IL-1β, IL-6, and TNF-α cytokines increased in patients bearing hip prosthetics after infection with coagulase-negative staphylococci [57]. The level of inflammatory cytokines IL-10 and TNF-α increased when biomaterials were infected with *Staphylococcus epidermidis* or *Pseudomonas aeruginosa* [58]. The current study observed a decrease in the systemic expression of IL-2 with single or dual species infections with or without implants. Other clinical and animal studies reported enhanced serum levels for IFN-γ, TNF-α, and IL-10, as well as decreased IL-2 levels in patients with *A. actinomycetemcomitans-* and *P. gingivalis-*associated periodontitis [59]. The present study systemically observed an increase in the expression of neutrophil chemoattractant CXCL1/Gro- upon single species implant infections, which was observed to increase in clinical studies and animal models during active bacterial infections [60,61,62,63].

Since cultural, immunologic, or DNA probe techniques have explained that several species of the oral microbiome interact with each other for the development of complex microbial biofilms [64]. Mixed species infections could trigger aggressive inflammation and rapid tissue loss in mice; therefore, complicated dual species bacterial infections were performed by mixing initial biofilm colonizers *S. oralis* and *A. actinomycetemcomitans* with late biofilm colonizers *P. gingivalis* and *T. denticola* [65]. Interestingly, and in agreement with observations documented from in vivo imaging of interferon-β in living mice, systemic levels of interleukins, chemokines, and growth-stimulating factors decreased upon dual species infections compared to single species infections. As indicated in Table 1, infection with *A. actinomycetemcomitans* as a single species caused a significant increase in IL-2, CXCL1, CCL-3/MIP1α, and G-CSF levels compared to sterile implants. *T. denticola* infection as single species on implants caused significant decrease in IL-2 expression compared to sterile implant. However, dual species infections with the mixture of *A. actinomycetemcomitans* and *T. denticola* significantly decreased IL-2, CCL-3/MIP1α, and G-CSF expression levels compared to sterile implants. Dual species infection with the combination of *A. actinomycetemcomitans* and *T. denticola* in the presence of implant material significantly decreased the expression of cytokines compared to tissue infection without implants. *S. oralis* infections as single species caused a significant increase in the expression of IL-1α, IL-6, IL-12 (p40), CXCL1/KC, CCL5/RANTES, and CCL11 compared to infection without implants. Interestingly, dual species infections with *P. gingivalis* and *S. oralis* decreased the expression of IL-6, IL-12 (p40), and CCL5/RANTES compared to tissue without implants. This observation can be explained since *P. gingivalis* antagonizes inflammatory response to other periodontal pathogens particularly because of its lipopolysaccharide (LPS) [66]. This has been already observed in human monocytes where co-stimulation with LPS from *P. gingivalis* and *Campylobacter rectus* sufficiently antagonized secretion of IL-6 and IL-8 [67,68]. In addition, *P. gingivalis* LPS prevented the apoptosis of neutrophils and countered the interaction of LPS from *Escherichia coli* for the regulation of inflammatory reactions in cells [69,70]. Decrease in cytokine induction mainly seemed to be arising from *P. gingivalis* or *T. denticola* since it was found that dual species *P. gingivalis–T. denticola* infections decreased IL-6, IL-1β, and CCL5/RANTES compared to single species infections with each of the bacterium in macrophage/epithelial cell co-culture model [71]. It can be speculated that inter-bacterial interactions that frequently occur in pathological oral biofilms have the capacity to undermine critical components of host immunity, including receptors, subsequent intracellular signaling pathways, and cytokine inductions. Therefore, it is proposed that dual species implant infections decrease the expression of inflammatory cytokines to promote prolonged persistence of pathogenic biofilm communities and to cause chronic inflammatory responses responsible for tissue destruction [72]. The overview of cytokine modification with dual species infections shows an exciting pattern and underlines the validity for preclinical studies regarding multispecies infection. Overall, implant-related infections by periodontal pathogens modulate the systemic expression of inflammatory cytokines. Such a modulation in inflammatory cytokines is suggestive of a challenge for the treatment of biomaterial-related bacterial infections that resist host immunity and conventional antibiotics therapy.

## 5. Conclusions

This study described the status of inflammatory cytokines in fibroblasts and then systemically studied them in mice with chronic peri-implant inflammation after infections with four different periodontal pathogens (alone or in combination). Fibroblasts were sensitive to bacterial co-cultivation and could only express five cytokines, suggesting that this in vitro experiment was insufficient to investigate complex bacteria–tissue interactions. The systemic modulation in the expression of inflammatory cytokines was specific to the type of pathogen, dependent on the presence of implants, and decreased with dual species infections. This suggests that bacterial infections as dual species decrease systemic cytokine expression to hide from the host inflammatory events. Such systemic modulation in the inflammatory cytokines reflects additional challenges associated with the treatment of biomaterial-associated infections. Moreover, these findings suggest the inclusion of multispecies to investigate biomaterial-related diseases for future animal models. Results from this sub-cutaneous model encourage extending this investigation to analyze inflammatory cytokines in future mouse models involving the implantation of infected implants inside bones in the oral cavity.

## Figures and Tables

**Table 1:** Effect of the single species or dual species bacterial infections around implants and status of inflammatory cytokine secretion. Symbols ***, **, *, and # indicate *p* < 0.001, *p* < 0.01, *p* < 0.05, and significant *p*-value prior to Bonferroni correction, respectively. (↑) indicate increase in the expression of cytokines, (↓) indicate decrease in the expression of cytokines.

Cytokines	Implant vs. So-Implant	Implant vs. Aa-Implant	Implant vs. Pg-Implant	Implant vs. Td-Implant	Implant vs. SoPg-Implant	Implant vs. AaTd-Implant	Importance in Peri-Implantitis
IL-1α					↓***		High expression in the manifestation of peri-implantitis [26]
IL-2	↓#	↑*		↓#	↓***	↓***	Higher levels in peri-implantitis [27]
IL-5			↓*		↓***	↓***	Expression in peri-implantitis was not significantly higher compared to healthy patients [28]
IL-6						↓**	The concentration remains higher in patients with peri- implantitis than in healthy implants [29]
IL-9						↓**	High expression not reported in peri-implantitis
IL-12 (p40)					↓***	↓***	High expression in peri-implantitis [30]
IL-12 (p70)	↓#		↓#	↓#	↓**	↓#	High expression in peri-implantitis [30,31]
IL-13			↓#		↓***	↓**	Antiresorptive agent to suppress osteoclastogenesis [32]
IL-17							Higher levels in peri-implantitis [33]
CXCL1/KC	↑**	↑#					Higher levels are found in periodontitis compared to healthy sites [34]
CCL5/RANTES			↓**		↓#	↓**	Pro-osteogenic: associated with macrophage transition from (M1) to (M2) phase [35]
CCL-3/MIP1α		↑#			↓#	↓#	Inflammation and bone resorption in periodontitis [36]; no statistically significant role reported thus far in peri-implantitis [37]
IFN-γ				↑#	↓**		IFN-γ considered as antiresorptive agents by suppressing osteoclastogenesis
G-CSF		↑#			↓#	↓#	No difference reported thus far in patients with peri-implantitis compared to patients with healthy implants [38]
TNF-α					↓#		Pro-inflammatory, promotes alveolar bone loss, augments production in peri-implantitis [39]
CCL2/MCP-1			↓#		↓***	↓***	Higher levels are found in periodontitis [34]; macrophages produce to promote fibrosis [40]
Upregulated	1	4	0	1	0	0	
Downregulated	2	0	5	2	12	11	

**Table 2:** Presence of implant material that play an important role in the systemic induction of inflammatory cytokines upon single or dual species infections. Symbols ***, **, *, and # indicate *p* < 0.001, *p* < 0.01, *p* < 0.05, and significant *p*-value prior to Bonferroni correction, respectively. (↑) indicate increase in the expression of cytokines, (↓) indicate decrease in the expression of cytokines.

Cytokines.	So-Implant vs. So	Aa-Implant vs. Aa	Pg-Implant vs. Pg	Td-Implant vs. Td	SoPg-Implant vs. SoPg	AaTd-Implant vs. AaTd
IL-1α	↑*		↑***		↓#	
IL-2	↓#	↑#				
IL-3		↑#				
IL-5		↑*				
IL-6	↑**		↓#	↑***	↓#	
IL-9			↓*			↑**
IL-12(p40)	↑#		↓**		↓***	↓#
IL-12(p70)						↓#
IL-13				↑#		
CXCL1/KC	↑#			↑#		
CCL5/RANTES	↑*				↓#	↓**
CCL-3/MIP1α		↑*		↑#		↓#
CCL11	↑#		↑#			
IFN-γ			↓#		↓*	
G-CSF					↓#	
CCL2/MCP-1		↑#		↑*		
Upregulated	6	5	2	5	0	1
Downregulated	1	0	4	0	6	4

## Data Availability

Not applicable.

## Supplementary Materials

The following supporting information can be downloaded at https://www.mdpi.com/article/10.3390/biomedicines10020286/s1, Figure S1: Classification of mice into respective groups for the measurement of cytokines. Figure S2: Expression of interleukins IL-6 and IL-12 in murine fibroblast. Figure S3: Systemic profiles of inflammatory cytokines 21 days post-implantation. Systemic cytokine expressed in the concentration of 0–10 pg/mL for IL-1α (A), IL-2 (B), IL-3 (C), IL-5 (D), IL-6 (E), and CCL3/MIP1α (F) were determined in the blood serum of mice carrying infected implants. Table S1: List of cytokines in pictograms per microliter (pg/mL) expressed near or below detection limits. (biological replicates (*n* = 3), technical replicates (3 each)). Table S2: Dual vs. single species infections: cytokine expression decreased with dual species infections compared to single species infections.
