# Supplementary material for: Bacterial-Specific Induction of Inflammatory Cytokines Significantly Decreases upon Dual Species Infections of Implant Materials with Periodontal Pathogens in a Mouse Model
Source: Biomedicines. 2022 Jan 26;10(2):286. doi: 10.3390/biomedicines10020286 (PMC8869624; doi:10.3390/biomedicines10020286)
Supplement: Supplementary file 1 [file biomedicines-10-00286-s001.zip › biomedicines-1526895-supplementary.pdf]

**SUPPLEMENTARY INFORMATION**

# Bacterial-specific induction of inflammatory cytokines significantly decreases upon dual species infections of implant materials with periodontal pathogens in a mouse model

Muhammad Imran Rahim[1*], Andreas Winkel[1], Alexandra Ingendoh-Tsakmakidis[1], Stefan Lienenklaus[2], Christine S. Falk[4], Michael Eisenburger[1], Meike Stiesch[1]

[1]Department of Prosthetic Dentistry and Biomedical Materials Science, Lower Saxony Centre for Biomedical Engineering, Implant Research and Development (NIFE), Hannover Medical School, Hannover, Germany

[2]Institute of Laboratory Animal Science, Hannover Medical School, Hannover, Germany

[3]Department of Orthopedic Surgery, Lower Saxony Centre for Biomedical Engineering, Implant Research and Development (NIFE), Hannover Medical School, Hannover, Germany

[4]Institute of Transplant Immunology, Hannover Medical School, Hannover, Germany

Corresponding Author

[*]Dr. Muhammad Imran Rahim
Department of Prosthetic Dentistry and Biomedical Materials Science
Hannover Medical School, Hannover, Germany
Email: Rahim.Muhammad@mh-hannover.de

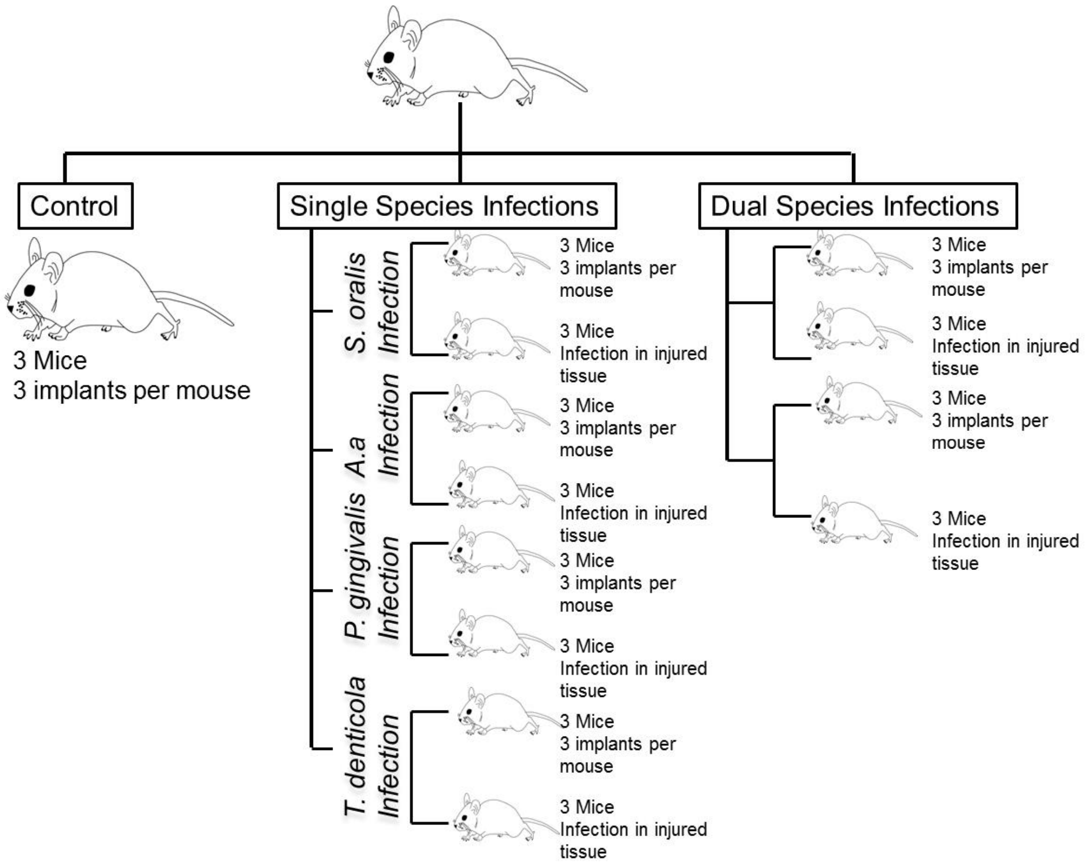


**Figure S1.** Classification of mice into respective groups for the measurement of cytokines.

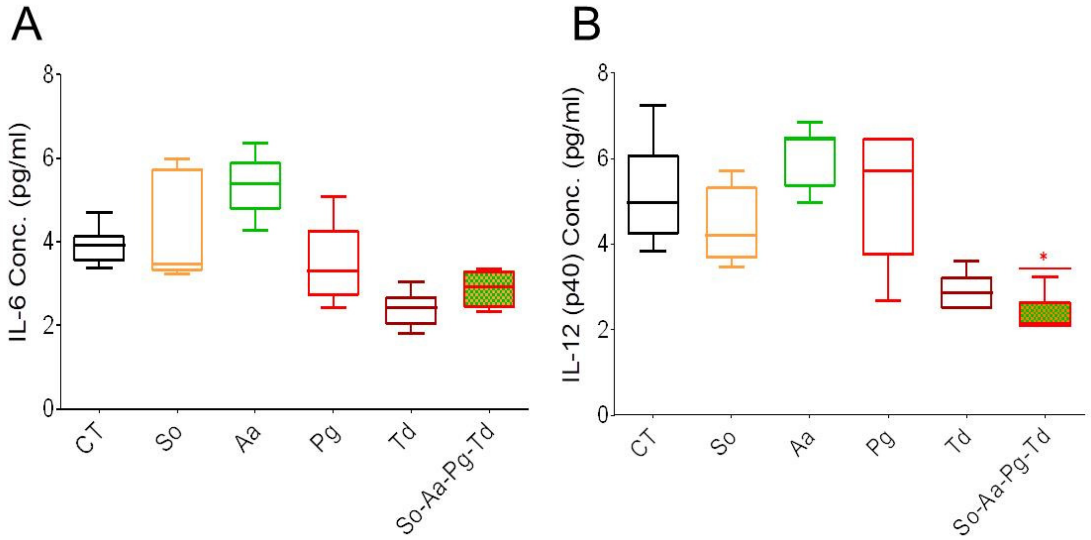


**Figure S2.** Expression of interleukins IL-6 (A) and IL-12 (B) in murine fibroblast.

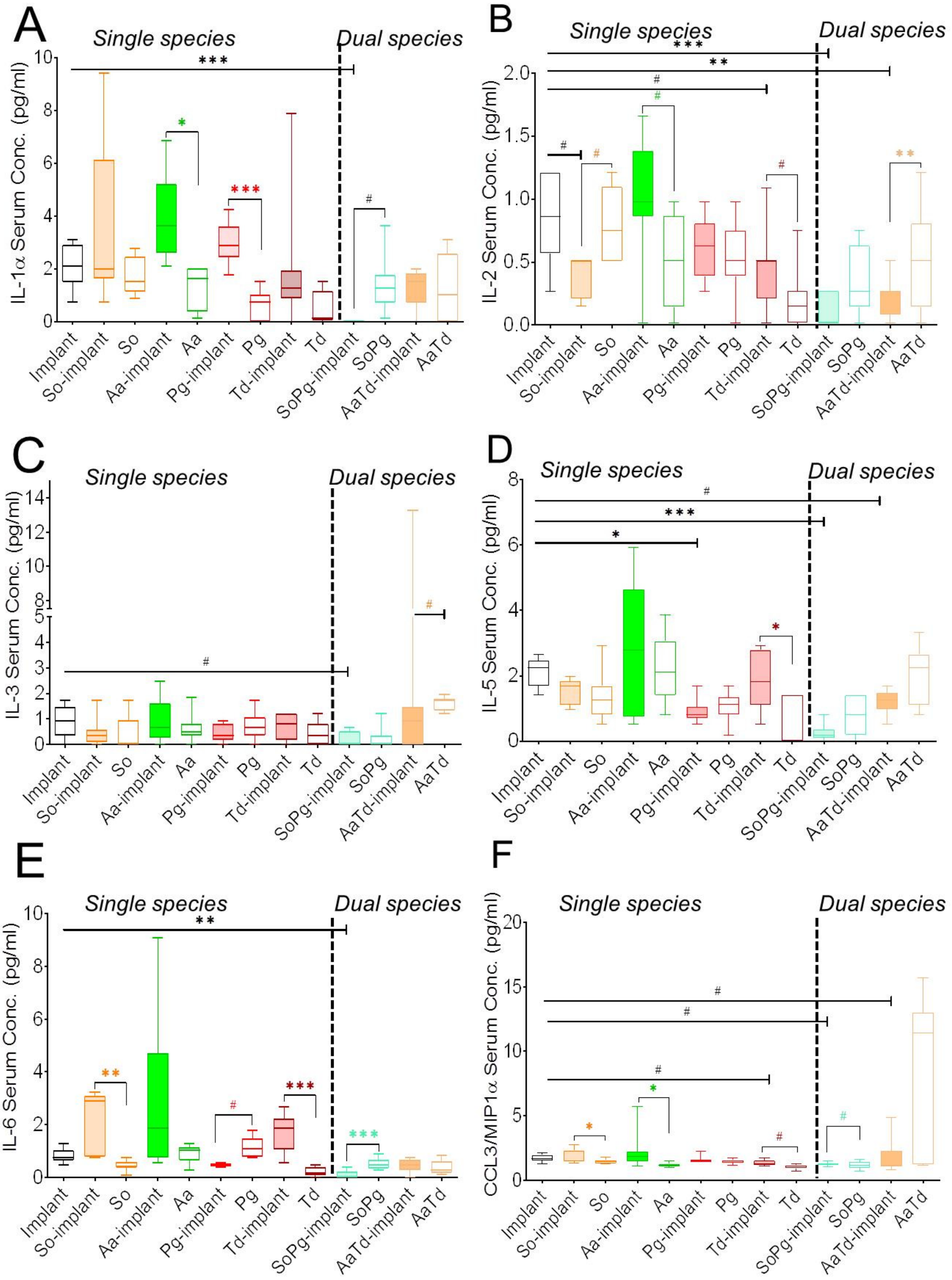


**Figure S3.** Systemic profiles of inflammatory cytokines 21 days post-implantation. Systemic cytokine expressed in the concentration of 0-10 pg/mL for IL-1$\alpha$ (A), IL-2 (B), IL-3 (C), IL-5 (D), IL-6 (E), CCL3/MIP1$\alpha$ (F) were determined in the blood serum of mice carrying infected implants.

**Supplementary Table S1.** List of cytokines in pictograms per microliter (pg/ml) expressed near or below detection limits. (biological replicates (n=3), technical replicates (3) each)

| | IL-1b | IL-4 | IL-10 | GM-CSF |
|---|---|---|---|---|
| Sterile implant | 0.57 | 0.27 | 2.92 | - |
| | 0.57 | 0.27 | 0.83 | |
| | 0.57 | 0.09 | 2.92 | |
| | 1.99 | | 0.83 | |
| implant + *S. oralis* | 0.57 | 0.45 | 0.83 | - |
| | 0.57 | | 2.92 | |
| | 3.15 | | | |
| | 0.57 | | | |
| | 1.99 | | | |
| *S. oralis* infection | 0.57 | 0.45 | 2.92 | - |
| | 0.57 | | | |
| | 0.57 | | | |
| | 0.57 | | | |
| Implant + *A. actinomycetemcomitans* | 1.99 | 0.62 | 6.51 | - |
| | 3.15 | 1.31 | 0.83 | |
| | 2.59 | 2.99 | 3.86 | |
| | | 0.45 | 4.77 | |
| | | | 0.83 | |
| *A. actinomycetemcomitans infection* | 0.57 | - | 0.83 | - |
| | 0.57 | | | |
| Pg-implant | 0.57 | 0.27 | 6.51 | - |
| | 1.99 | 0.27 | 6.51 | |
| | | | 0.83 | |
| | | | 2.92 | |
| | | | 4.77 | |
| | | | 1.92 | |
| Pg | 0.57 | - | 0.83 | - |
| | | | 0.83 | |
| Td-implant | 0.57 | 1.14 | - | - |
| Td | - | - | 4.77 | - |
| SoPg-implant | - | - | 0.83 | - |
| | | | 0.83 | |
| | | | 2.92 | |
| SoPg | - | - | 0.83 | - |

| | | | | |
|---|---|---|---|---|
| AaTd-implant | 0.57 | 1.48 | 0.83 | 7.76 |
| AaTd | - | 1.90 | 0.83 | 10.68 |
| | | 1.48 | 2.92 | 1.13 |
| | | 1.65 | 6.51 | 7.76 |
| | | 1.31 | 2.92 | 14.58 |
| | | | 8.17 | 9.77 |
| | | | 2.92 | |

**Supplementary Table S2.** Dual vs single species infections: Cytokine expression decreases with dual species infections compared to single species infections

| Cytokines | SoPg vs So | SoPg vs Pg | SoPg-implant vs So-implant | SoPg-implant vs Pg-implant | AaTd- vs Aa | AaTd- vs Td | AaTd-implant - vs Aa-implant | AaTd-implant- vs Td-implant |
|---|---|---|---|---|---|---|---|---|
| IL-1$\alpha$ | | | ↓*** | ↓*** | | | ↓* | |
| IL-2 | ↓# | | | ↓* | ↑# | | | ↓*** |
| IL-3 | | ↓# | | | ↑# | ↑** | | |
| IL-5 | | | | ↓* | | ↑** | | |
| IL-6 | | ↓# | ↓*** | ↓# | | ↓# | ↓* | ↓* |
| IL-9 | | ↓*** | ↓# | ↓# | | ↑* | | |
| IL-12(p40) | ↑# | ↑*** | ↓** | ↓*** | | | ↓# | ↓* |
| IL-12(p70) | | ↓# | | | * | | ↑ | |
| IL-13 | | | ↓# | ↓# | | ↓# | ↓# | ↓# |
| CXCL1/KC | | | ↓*** | | ↓* | | ↓# | |
| CCL5/RANTES | ↑# | ↑* | ↓# | | | ↑** | ↓# | ↓# |
| CCL-3/MIP1$\alpha$ | | ↓# | ↓# | ↓# | ↑** | ↑*** | ↓# | |
| CCL11 | | | ↓# | | | | | |
| IFN-$\gamma$ | | ↓*** | ↓# | ↓# | | ↑** | | |
| G-CSF | | | | ↓* | ↓# | | ↓*** | ↓** |
| TNF-$\alpha$ | | ↓# | | | | ↑* | | |
| CCL2/MCP-1 | ↓* | | ↓*** | ↓# | | | ↓# | ↓# |
| Upregulated | 2 | 2 | 0 | 0 | 3 | 8 | 0 | 0 |
| Downregulated | 2 | 7 | 11 | 11 | 2 | 2 | 9 | 7 |

Statistically significant increase (↑) or decrease (↓) in the serum cytokine levels at ***, **, *, and # indicated $p < 0.001$, $p < 0.01$, $p < 0.05$, and significant $p$ value prior to Bonferroni correction, respectively, between implants and infected implants in mice upon single or dual species infections.
